# Invasive Intraneural Interfaces: Foreign Body Reaction Issues

**DOI:** 10.3389/fnins.2017.00497

**Published:** 2017-09-06

**Authors:** Fiorenza Lotti, Federico Ranieri, Gianluca Vadalà, Loredana Zollo, Giovanni Di Pino

**Affiliations:** ^1^NeXT: Neurophysiology and Neuroengineering of Human-Technology Interaction Research Unit, Università Campus Bio-Medico Rome, Italy; ^2^Research Unit of Orthopaedic and Trauma Surgery, Università Campus Bio-Medico Rome, Italy; ^3^Fondazione Alberto Sordi-Research Institute for Aging Rome, Italy; ^4^Research Unit of Neurology, Neurophysiology and Neurobiology, Università Campus Bio-Medico Rome, Italy; ^5^Research Unit of Biomedical Robotics and Biomicrosystems, Università Campus Bio-Medico Rome, Italy

**Keywords:** foreign body reaction, invasive neural interface, peripheral nerve stimulation, intraneural electrodes, neural interfaced prostheses

## Abstract

Intraneural interfaces are stimulation/registration devices designed to couple the peripheral nervous system (PNS) with the environment. Over the last years, their use has increased in a wide range of applications, such as the control of a new generation of neural-interfaced prostheses. At present, the success of this technology is limited by an electrical impedance increase, due to an inflammatory response called foreign body reaction (FBR), which leads to the formation of a fibrotic tissue around the interface, eventually causing an inefficient transduction of the electrical signal. Based on recent developments in biomaterials and inflammatory/fibrotic pathologies, we explore and select the biological solutions that might be adopted in the neural interfaces FBR context: modifications of the interface surface, such as organic and synthetic coatings; the use of specific drugs or molecular biology tools to target the microenvironment around the interface; the development of bio-engineered-scaffold to reduce immune response and promote interface-tissue integration. By linking what we believe are the major crucial steps of the FBR process with related solutions, we point out the main issues that future research has to focus on: biocompatibility without losing signal conduction properties, good reproducible *in vitro*/*in vivo* models, drugs exhaustion and undesired side effects. The underlined pros and cons of proposed solutions show clearly the importance of a better understanding of all the molecular and cellular pathways involved and the need of a multi-target action based on a bio-engineered combination approach.

## Introduction

**Figure 1:**
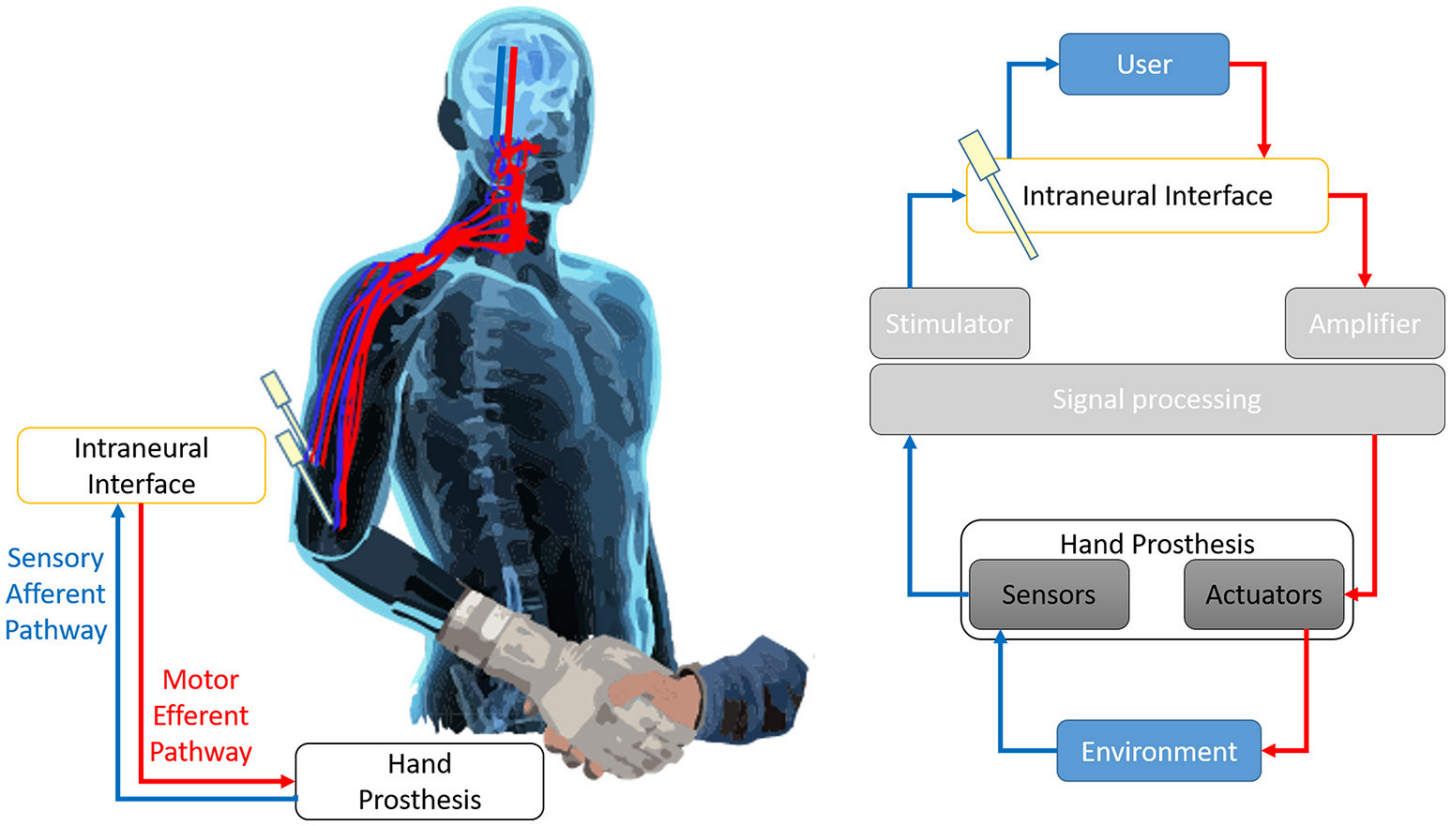
Prosthesis closed loop control.The continuity of the efferent motor pathway (in red) and the sensory afferent pathway (in blue) creates a double bidirectional connection between the User and the prosthesis. The prosthesis acts on, and experiments, the environment. In such way it is established a closed loop control that allows to have an almost real-time feedback of the effects of the motor commands and to correct them accordingly. The Invasive intraneural interface is the crucial component puts along both arms of the loop, since it mediates the information flow from the brain to the actuators of the prosthesis and from the sensors embedded on the prosthesis back to the brain. The Figure represents the sensorimotor loop superimposed on the body of the User **(left)**, and a chart of the main components of the loop and their interrelation **(right)**.

The loss of a limb after amputation, altering motor and sensory functions, severely affects the daily lives of patients (Pasquina et al., 2006). Today's Robotics is able to offer sensorized mechatronic hands with amazing performance; to these devices, conventional body-powered or electromyographic control systems revealed to be inadequate. In order to overcome such issue, novel advanced neural prosthetic devices, which attempt to interface bidirectionally with the user through invasive microelectrodes implanted inside stump's peripheral nerves (Di Pino et al., 2013), are in developmental phase (Di Pino et al., 2009). Basically, these neural interfaces are used to deliver afferent information to the nervous system (sensory feedback) (Benvenuto et al., 2010; Di Pino et al., 2012; Raspopovic et al., 2014), and at the same time, to extract consciously-modulated neuronal signals (motor volition) from healthy portions of the nervous system (Figure 1). Then, through a variety of signal processing algorithms, these signals can be used to drive an external device, such as a prosthesis (Rossini et al., 2010). Such interfaces have the potential to bring crucial advancements to the user: (a) to get a more complex signal, compared to the muscular one, allowing to control more degrees of freedom of the prosthesis; (b) to transmit the sensory information to the central nervous system (CNS) allowing to feel and experience the environment; (c) to restore the original closed “sensory-motor loop” control, meaning to have prosthesis and user linked by a double connection where information can travel from one to the other in both directions. A closed loop control allows to accurately exploit in real time sensory information to drive motor command, while having the feed-back of the outcome of the command and constantly readjust it to reduce any deviation from the desired targeted action.

When developing new neural interfacing devices, the attempt of the researchers is, on one hand, to preserve some important features, such as biocompatibility and selectivity properties, and on the other hand, to minimize the correlated potential counterpart, such as immune response reaction, the invasiveness and the risks of the implanting procedure. In this review, we focus our attention on biological challenges, mainly because all the efforts to develop a more immune system-friendly recording/stimulating device will also prevent the complete loss of functionality over time and, in turn, the loss of all the improvements done for reaching high selectivity and implantable ease.

## The foreign body reaction

The placement of any therapeutic biomaterial-based device in the *in vivo* environment requires surgical actions that damage the target tissues (Anderson, 1988). Following those damages, all the processes to restore tissue homeostasis around the implant are part of the physiological regeneration called wound healing (Teller and White, 2009).

However, the continuous presence of any medical implants contributes to a long-term overstimulation of the immune system, which leads to chronic inflammation and poor wound healing. This unbalanced reaction, also known as the foreign body response or reaction (FBR), determines the implant failure and might have contributed to implant reduction functionality in human (Dhillon et al., 2005; Rossini et al., 2010; Horch et al., 2011; Raspopovic et al., 2014) and animal trials (Lago et al., 2007b; Badia et al., 2011; Groothuis et al., 2014; Harreby et al., 2015; Wurth et al., 2017), due to the formation of a cellular, protein mediated, capsule around the implant.

**Figure 2:**
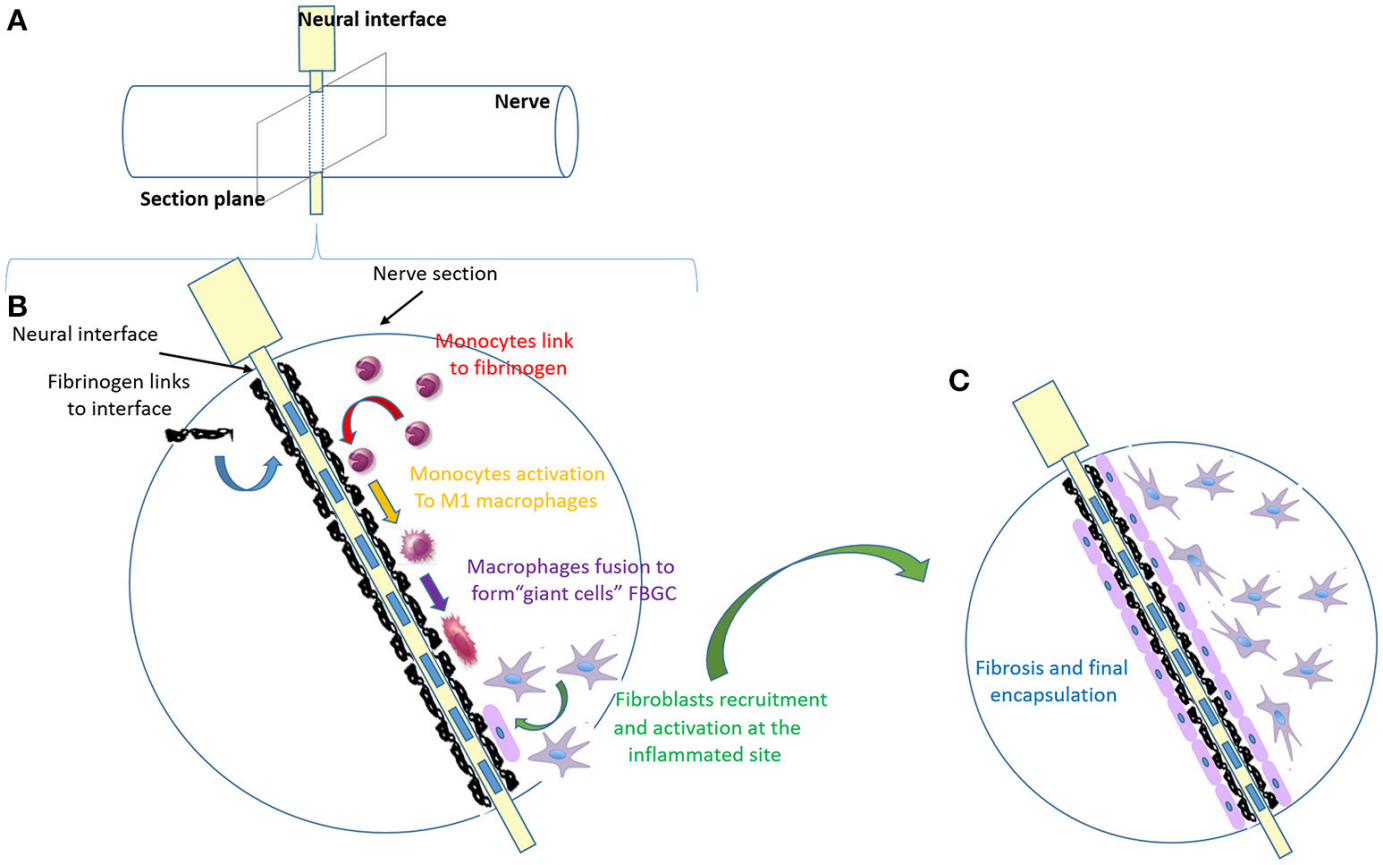
Major FBR process steps in a nerve implanted with a neural interface. **(A)** Schematic image of the electrode (in yellow) inserted within the nerve (in white). **(B)** Zoom in of the nerve section, as in **(A)**, implanted with the electrode (in white) with the metallic active contacts embedded (blue rectangles). This panel resumes the main steps of FBR over time, which are shown with different colors. Step 1 (in black): blood-plasma proteins adsorption to the foreign body; step 2 (in red): monocytes recruitment and differentiation into macrophages; step 3: macrophages activation (yellow) and fusion to form giant cells (purple); step 4 (in green): fibroblasts recruitment and activation to form fibrotic tissue. **(C)** Final stable encapsulation of the electrode with fibrotic tissue, where fibroblasts (in purple) form a compact dense tissue.

While it has been suggested that encapsulation is beneficial in interfaces, such as epimysial and cuff electrodes as it stabilizes the implant (Grandjean and Mortimer, 1986; Grill and Mortimer, 1998), FBR is an important issue to be solved in intraneural electrodes (Navarro et al., 2005), limiting device function over time, often forcing a premature removal (Badia et al., 2011). We can divide the FBR process in four major steps: (1) blood-plasma proteins adsorption to the foreign body; (2) monocyte recruitment and differentiation to macrophages; (3) macrophages activation and fusion to form giant cells; (4) fibroblasts recruitment and activation to form fibrotic tissue (Figure 2). Regardless of the tissue or organ into which a biomaterial is implanted, the initial inflammatory response is activated by injury to vascularized connective tissue. Immediately after implantation (step 1), proteins including fibronectin, vitronectin, albumin, fibrinogen and complement factors derived from blood-plasma will adsorb to biomaterial surfaces, generating a blood-based barrier that causes thrombosis, guides the movement of monocytes and neutrophils to implant surface and activates complement and coagulation cascade (Szaba and Smiley, 2002). Monocyte recruitment and differentiation to macrophages (microglia in CNS) (step 2) may continue up to weeks, and chemotactic factors are released over longer periods of time (Tang and Eaton, 1999). Plasma fibronectin, among all plasma proteins, after coating the implant surface, changes its conformation and seems to act specifically as receptor for fibroblast and additional macrophages (Keselowsky et al., 2007). This persistent inflammatory stimulus leads to chronic inflammation confined to implant site. More in details, the cytokine release (e.g., IL-4 and IL-13; IL-1β, and TNFα in CNS) from the microenvironment (Shen et al., 2004) leads to the fusion of the accumulated macrophages to form foreign body giant cells (FBGC) (step 3) (Anderson, 2000). Original purpose of FBGC formation, producing catabolic enzymes and acids, is to try to digest the foreign body; its side effect is the possibility of causing implant damage. Macrophages are probably the most important cells in chronic inflammation, because of the great number of biologically active products they produce, including multiple growth factors that are extremely important to promote: (i) further recruitment of monocytes/macrophages cell population; (ii) growth of fibroblasts and their differentiation in myofibroblasts (astrogliosis in CNS); (iii) blood vessels and epithelial cells regeneration (Anderson et al., 2008). The proliferation of myofibroblasts (step 4) in developing granulation tissue leads to active synthesis of extracellular matrix (ECM) components, such as collagen and proteoglycans to form the fibrous capsule, which is an attempt to isolate the foreign material from local tissue environment (de Fougerolles and Koteliansky, 2002; Diegelmann and Evans, 2004; Ratner and Bryant, 2004). The degree and extent of the macrophages (microglia in CNS) activation, leading to FBR at the implant site, is dependent on surface properties of the biomaterial (porosity, roughness, stiffness, and chemistry), the shape of the implant, the relationship between the surface area of the biomaterial and the volume of the implant. Thus, FBR may be likely controlled by acting on those factors. The efforts put on biomaterial studies to develop a more biocompatible device have led to understand some important FBR-modulating characteristics applied to build the more recent neural interfaces: (i) the acute response is proportional to the diameter of the interface, so smaller implant size elicits less fibroblasts (astrocytes) reactivity (Szarowski et al., 2003; Veiseh et al., 2015); (ii) the adhesion of serum components and macrophages (microglia) is decreased on hydrophilic materials (Leung et al., 2008); (iii) shape without sharp corners allows for a mild mechanical trauma insertion (Groothuis et al., 2014; Veiseh et al., 2015); (iv) material requires a degree of stiffness able to give the interface the right strength to bear the tissue tension and, at the same time, the flexibility to reduce the mismatch with the surrounding tissue (Schoen and Anderson, 2004; Moshayedi et al., 2014); (v) electrical contact symmetrically distributed along the interface contribute to a uniform current distribution (Harnack et al., 2004; Groothuis et al., 2014); (vi) roughness effects are still very unpredictable since they are cell-specific and dependent on the material and porosity (Christo et al., 2015).

### Intraneural electrodes for peripheral nervous system (PNS)

This review is focused on peripheral nervous system (PNS) interfaces as they are a very attractive functional interface, and represent a good approach to study nerve system stimulation and recording. Compared to the CNS, the benefits coming from working on the peripheral nerve is a reduced invasiveness and the opportunity of using the motor and sensory fibers, both within the nerves, to create a bidirectional communication. Depending on nerve invasiveness, different types of electrodes have been developed to interface the PNS. Here we focus on intraneural electrodes because, if FBR-related biological issues will be addressed, they represent a good compromise between invasiveness and selectivity, so probably the best interface choice. Intraneural electrodes, such as LIFEs (longitudinal intrafascicular electrodes) (Yoshida and Horch, 1993; Lawrence et al., 2004), their evolution tfLIFEs (thin-film longitudinal intrafascicular electrodes) (Lago et al., 2007a), TIMEs (transverse intrafascicular multichannel electrodes) (Boretius et al., 2010), SELINE (self-opening intrafascicular neural interface) (Cutrone et al., 2015) are implanted within nerve fascicles, which leads to higher nerve fibers selectivity, lower axons stimulation threshold and better signal recording (Badia et al., 2011). Utah Slant Electrode Array (UEA) are arrays composed by tens of different length needles (Nordhausen et al., 1996) able to reach more fascicles. A more recent version of these arrays (Lacour et al., 2010) has a less rigid structure that can be deformed along with the nerve, allowing for a less trauma and tension at the interface and a lower risk for the nerve to be damaged. However, from the few experiments performed so far on PNS, concrete advancements compared to the other intraneural electrodes, in terms of long-term signal stability, cannot be gathered (Warwick et al., 2003; Branner et al., 2004). Compared to other implant devices, neural interfaces have the peculiarity to deliver current. As discuss later, it is still a matter of debate whether this has a relevant effect on FBR development.

### Consequences of the FBR on intraneural electrode performance

The fibrotic encapsulation occurring at the end of the FBR and the whole inflammatory response with the related increase of impedance at the electrode contacts, imposing continuous adjustments of stimulation parameters in terms of intensity and duration, seem to be the main causes of electrode failure in intraneural chronically-implanted devices (Lago et al., 2005; Kundu et al., 2014). In order to have intraneural electrodes applied in patients, as a part of a new prosthesis generation and even prospective wider applications (Di Pino et al., 2014), it is important to assess the electrical stability by monitoring performance over time. Unfortunately, this goal has not been achieved so far, due to the difficulties in studying the relative roles of cytokines and cell-material interactions during the inflammatory phases *in vivo*, the lack of their specific control and the wide range of experimental variables coming into play. Evidence of the electrical failure associated with FBR came from the wider experience on cortical electrodes implantation. In rat, it has been shown that electrodes elicit a significant FBR in terms of reactive gliosis which, in turn, changes also their impedance spectrum (Williams et al., 2007; Lempka et al., 2009; McConnell et al., 2009), confirming *in vivo* what has been found in a 3D culture of astrocytes and microglia encapsulating an electrode (Frampton et al., 2010). It has been also shown a correlation between the increased impedance of chronically implanted epineural electrodes in rats and the growth of fibrous tissue around the electrode contacts (Murphy et al., 2004). In cat brain, the electrical insulation, caused by the fibrotic encapsulation, affected electrode recording quality among different session (Schultz and Willey, 1976; Liu et al., 1999), as well as electrical impedance (Roitbak and Sykova, 1999). In the perspective of a long-term implantation, the functional consequence is that more reactive gliosis leads to higher impedance and requires higher stimulation intensity, in terms of delivered charge (Brown et al., 1977; Butson et al., 2006), which may even become not compatible with the *in vivo* applications. Routinely, tissue-component measurements of the impedance at the electrode implant site using impedance spectroscopy, has been proposed as a tool to monitor the proportional development of fibrous tissue, giving the researchers the possibility to check FBR over time and intervene meanwhile. The formation of fibrotic sheath is not the only FBR aspect that causes electrode failure, but tissue-electrode mismatch and micro-motion or nerve damage with neural cell loss also give a contribution (Groothuis et al., 2014). Longitudinal intra-fascicular Pt-LIFE and tfLIFE electrodes have been implanted in the sciatic nerve of rats (Lago et al., 2007b) and TIME electrodes have been also implanted in pig median nerve (Badia et al., 2011; Kundu et al., 2014; Harreby et al., 2015). In all cases, it has been found just a mild fibrous scar around the implant, increasing over time, with the presence of infiltrating macrophagic cells. Macrophages indicate an ongoing inflammatory reaction for up to 90 days, without any signs of axonal loss or degeneration. Stimulation tests demonstrated that the conduction velocity and amplitude of muscle response decreased in the first 4 weeks; however, the functional responses normalized during the following months. Recently it has been observed, up to 6 months, a specific correlation between the thickness of the fibrotic capsule around the implant in rat sciatic nerve and the stimulation threshold and electrode impedance increase. Moreover, it seems that electrical stimulation does not primarily contribute to enhance the FBR, suggesting that insertion trauma and chemical/mechanical mismatch represent the major players in the process (Wurth et al., 2017). Studies on humans implanted with intraneural electrodes have reported an increasing amount of electrical charge to be delivered over 4 weeks to evoke the same response after median nerve stimulation (Dhillon et al., 2005; Rossini et al., 2010; Horch et al., 2011; Raspopovic et al., 2014). These were the only data obtained, since the clear impossibility of matching the morphological evaluation of the tissue around the implant site with the hypothesis of an ongoing FBR. Although these studies have evaluated the intraneural electrode in long-term implants, they did not assess all the microscopic tissue-electrode changes following implantation.

## Possible strategies to reduce FBR

There are many ways by which it is possible to interfere with the FBR, but none of them seemed to solve the issue. Thus, we think it is mandatory to elucidate molecular and cellular pathways involved in the host immune response triggered by the electrode, and how drugs, co-factors and biomaterials can affect them. However, this kind of studies is very challenging due to the lack of a good, reproducible *in vitro* model that recapitulates what physiologically happens in the nerve tissue architecture. The 3D *in vitro* model developed so far to quantify inflammatory response to biomaterial might be a starting point to reproduce an immune system cells environment within the nervous system (Almeida et al., 2014; Parks et al., 2014). Other challenges are present also in the *in vivo* model, meaning inter-species variabilities and the difficulties of monitoring the roles of each cellular component and cytokine network involved in FBR. Moreover, all the issues related to how to specific deliver, locally or systemically, any kind of bio-active anti-inflammatory substance over the time should be analyzed. This review highlights the development of strategies with regulating effect on the FBR, with important implications for PNS stimulation and for the possibility to create implanted long-term functional medical devices. We propose a systematic approach for FBR by linking each step of the FBR process (i.e., blood and plasma proteins adsorption to the foreign body; monocyte recruitment and differentiation to macrophages and their fusion to form giant cells; fibroblasts recruitment and activation to form fibrotic tissue) with possible solutions. These solutions, of which pros and cons are underlined, are a selection, from literature and the related research field, of what we think is the most promising strategy. Based on these premises, we focus on and provide two main directions to overcome FBR and promote interface stability: (1) acting on the interface properties and (2) acting on the interface-microenvironment interaction.

### Acting on the interface itself

**Figure 3:**
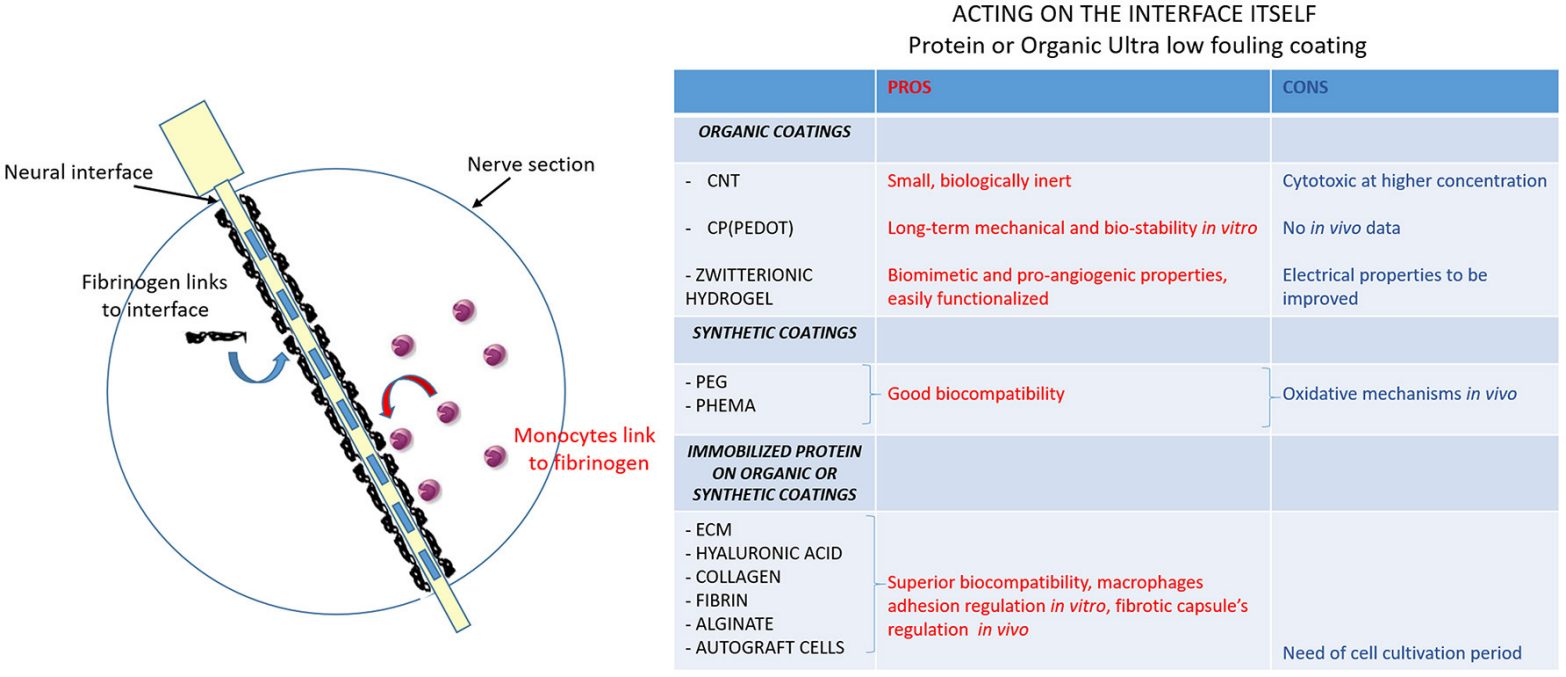
Ultra low fouling coatings to modulate protein adsorption to the interface. Schematic image of nerve section implanted with the electrode. The first two steps of the FBR are shown: blood-plasma proteins (in black: fibrinogen mainly) adsorption to the electrode (in yellow with metallic contacts as blue rectangles) and monocytes recruitment at the fibrinogen site and differentiation into macrophages. The table resumes the actions that can be done to modify electrode surface. The creation of ultralow fouling neural interface coatings can modulate monocytes-fibrinogen interaction: pros (red column) and cons (blue column) of organic coatings, synthetic coatings and immobilized protein on organic and synthetic coatings are evaluated.

This means to modify shape and material of electrodes, including the use of immobilized protein coatings or organic coatings (Figure 3). Most of the novel intraneural electrodes are made of a polyimide core with metallic tracks, typically platinum (Pt), platinum-iridium (Pt-Ir) and gold (Brummer et al., 1983; Geddes and Roeder, 2003). Those metals seem to be the best choices for stimulating electrodes due to their electrochemical stability, poor biological reactivity and corrosion resistance (Merrill et al., 2005; Polikov et al., 2005).

Because nonspecific absorption on the device surface is the main step triggering the FBR, acting on the non-fouling quality of the material may prevent the capsule formation. Carbon nanotubes (CNTs), conductive polymers (CPs), conductive hydrogels (CHs) are the latest materials under investigation for a stable neural interface (Aregueta-Robles et al., 2014). All of them are organic materials, making them easily bio-functionalized to increase biocompatibility and electrochemical surface area and, in turn, improve performance over time.

CNTs are small, biologically inert (Seidlits et al., 2008) and enhance electrical properties of the electrode (Green et al., 2008; He et al., 2011; David-Pur et al., 2014), however they are cytotoxic at high concentration (Bottini et al., 2006; Tian et al., 2006).

An innovative and promising material developed from CNT is a nanostructured boron-doped diamond (BDD) made of vertically aligned nanotubes template encapsulated in two BDD nanolayers. Such biomaterial seems to be well suited for neural interface since it combines the diamond electrochemical properties, providing good neural stimulation and recording performance, with the structural stability of the nano-structure. The concerns about the cytotoxicity were addressed by some *in vitro* and *in vivo* (subcutaneous implant up to 4 weeks) experiments that have shown good biocompatibility together with lower inflammation response and smaller fibrous capsule formation (Piret et al., 2015; Alcaide et al., 2016).

Poly (3,4-ethylenedioxythiophene) PEDOT and some of its modified versions, are the most promising CPs that have been evaluated, and they have been shown to improve electrochemical impedance (Kim et al., 2004, 2008; Abidian et al., 2010; Green et al., 2013) and charge injection limit as a result of the PEDOT roughness within a hydrated environment (Cui et al., 2003; Ravichandran et al., 2010; Green et al., 2013). Despite some promising results *in vitro* (Bolin et al., 2009; Evans et al., 2009; Green et al., 2009), there are no *in vivo* results supporting conductive polymers long-term mechanical and bio-stability (Wilks et al., 2011).

Poly (ethylene glycol) PEG (Drury and Mooney, 2003) and poly (2-hydroxyethyl methacrylate) PHEMA (Mario Cheong et al., 2014) synthetic hydrogels are the most widely low-fouling material used *in vivo*, but their long-term application is limited due to oxidative mechanisms partially reducing non-specific protein absorption.

Recently, a new class of ultra-low-fouling biomaterial, called zwitterionic hydrogels, are under consideration for their biomimetic and proangiogenic properties, and because they are very easy to be functionalized by incorporating bioactive molecules (Willerth and Sakiyama-Elbert, 2007), such as growth factors or drugs. Zwitterionic hydrogels, prepared from carboxybetaine, not only strongly resist the formation of the fibrotic capsule in a mouse model for at least 3 months, but they are also able to more effectively recruit pro-healing macrophages phenotype and enhance micro-vessels formation in the surrounding tissue (Zhang et al., 2013).

The issue that hydrogels do not improve electrical properties might be solved in the future by incorporating some conductive components, such as conductive polymers (CPs) like PEDOT, previously analyzed. Basically, the CP-hydrogels hybrids will join the mechanical stability and biocompatibility of the hydrogels with the enhanced electrical features of the CPs, resulting from their rough morphology and from the incorporation of some dopant ions, including poly-styrene sulphonate (PSS), paratoluene sulphonate (pTS), dexamethasone phosphate (Dex-P), and perchlorate (ClO_4_). There are some promising preliminary *in vitro* studies that need to be confirmed by successful *in vivo* performances (Green et al., 2013; Aregueta-Robles et al., 2014). Moreover, it is important to deeper understand how the two polymers integrate one another in order to control the system structure and function to meet the long-term electrode stimulation requirements.

In contrast to synthetics, organic biomaterials and hydrogels composed of ECM components, collagen (Suri and Schmidt, 2010), hyaluronic acid (Hsieh et al., 2014), fibrin (Ahmed et al., 2008), and alginate (Banerjee et al., 2009) represent a better choice, because they exhibit superior biocompatibility (Kim et al., 2007; Fujihara et al., 2010) and have been shown to effectively regulate the macrophage adhesion and activation *in vitro*, as well as the capsule formation *in vivo* (Hsieh et al., 2014). Previous studies have found that allogeneic human ECM proteins are well tolerated by the host and do not appear to elicit either a cell mediated or humoral immune response (Allaire et al., 1994, 1997). Moreover, in a futuristic picture, a patient own biopsied cells could also be used to create autograft material. However, to these days, the production of an autograft material would require preoperative cell harvest followed by a moderate cultivation period (weeks/months), limiting its use to clinical applications, only if time is not a critical factor.

### Short-term acting on the interface-microenvironment

This intervention includes coupling the implant with absorbable scaffolds (Chan and Leong, 2008) that make the local delivery of FBR blocking drugs (anti-inflammatory, anti-fibrotic) possible at high concentration, but for a period of time limited by scaffold degradation. Looking for a reduced FBR on the surface of the implants, the material chemistry has been optimized and functionalized to reduce protein deposition, the first step of FBR (Thevenot et al., 2008; Goodman et al., 2013). Therapeutic molecules, such as growth factors or anti-inflammatory drugs, have been embedded onto the implant surface for slow release into the tissue microenvironment (Couto et al., 2012; Goodman et al., 2013; Lerner and Dombrowski, 2015). However, they just seem to delay and not prevent the FBR.

#### Delivery of anti-inflammatory agents

**Figure 4:**
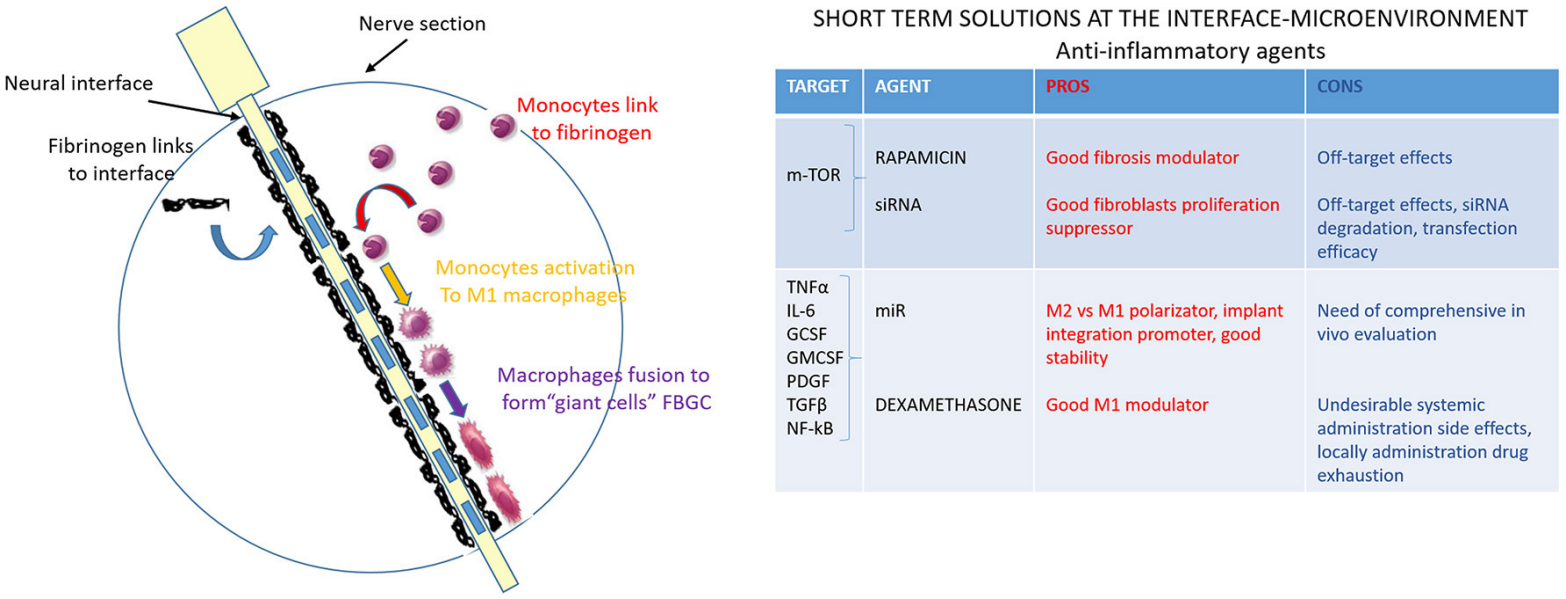
Anti-inflammatory agents to modulate the interface-microenvironment immune-mediated interaction. Schematic image of nerve section implanted with the electrode. The third step of the FBR is shown: monocytes are activated into pro-inflammatory macrophages M1 (in yellow) and they fuse together to form giant cells (in purple). Pro-inflammatory and cytotoxic soluble factors, secreted by reactive macrophages at the device-tissue interface, are the most likely mediators of the cellular changes underlying the FBR. The table resumes anti-inflammatory agents (drugs, siRNA, miR) that can modulate the interface-microenvironment immune-mediated interaction. Pros (in red) and cons (in blue) of acting on some mediators of pro-inflammatory pathways are summarized.

Pro-inflammatory and cytotoxic soluble factors secreted by reactive macrophages at the device-tissue interface, are the most likely mediators of the cellular changes underlying the FBR. Based on this assumption, electrodes developed in a way that can reduce macrophages activation, or the concentrations of their released soluble factors surrounding the implant, will affect the severity of the FBR. Some potential strategies to reduce FBR are decreasing the amount of device surface area for macrophage interaction/activation and, more intriguing, incorporating permeable coatings that release cytokines to improve clearance of macrophage-released factors (Figure 4). Based on work in which anti-inflammatory minocycline drug administration seemed to improve recording performance (Rennaker et al., 2007), probe coatings that locally released anti-inflammatory dexamethasone were developed (Zhong and Bellamkonda, 2007). While systemic dexamethasone administration was long-term effective but caused undesirable side effects (Zhong and Bellamkonda, 2007), dexamethasone coatings significantly reduced activated macrophages and improved impedance measurements (Spataro et al., 2005; Kim and Martin, 2006; Mercanzini et al., 2010) 1 week post-implantation in rat cortex. Unfortunately, this reduction effect was lost after 4 weeks, probably because of the drug source exhaustion. If this were the case, a chronic anti-inflammatory regimen might be needed to reduce the FBR through the whole lifetime of the implanted device.

Lots of drugs, extensively investigated as potent cell cycle regulators in cancer, may represent a good target to prevent also the fibroblasts hyperproliferation that occurs in FBR. Rapamycin, a specific mTOR inhibitor, has been reported to be effective in modulating *in vivo* fibrosis not only in cardiac and pulmonary context, but also in the FBR, as a key activator of collagen type I. A device-based local delivery of siRNA (small interfering RNA) against mTOR has been tested to inhibit fibrous encapsulation in a murine subcutaneous implant model (Takahashi et al., 2010). In general, targeting siRNA in a systemic disease represents a risk for pathological off-target effects, such as silencing unintended genes (Singh et al., 2011), but, in the context of the localized process of the FBR, this issue can be overcome with a polymer-based delivery system. Therefore, siRNA-releasing hydrogel-coated device has been used to suppress fibroblast proliferation and down-regulate type I collagen mRNA expression. Even if they were not able to reproduce *in vivo* the same good results got *in vitro*, the use of specific siRNA targeting fibroblasts, combined with the local delivery, remains an attractive therapy (Ozcan et al., 2015). Problems, such as siRNA degradation or better transfection efficacy, need to be solved in order to better exploit the siRNA specificity for a successful application in the FBR context. A better choice could be the use of chemically modified antisense miRs (microRNA). They have been proposed as good candidates to re-direct host immune response toward implant integration (Ong et al., 2015), in particular to promote anti-inflammatory polarization of adherent macrophages (M2), for example with IL-4, IL-10, IL-13 (van Putten et al., 2009; Sica and Mantovani, 2012). Compared to siRNA/shRNA, miRs are stable in blood plasma because they are normally secreted by immune system (Hunter et al., 2008; Mitchell et al., 2008; Weber et al., 2010) and they can act on different targets, exerting a wider effect on multiple pathways and biological mechanisms (Lim et al., 2005; Selbach et al., 2008).

#### Delivery of anti-fibrotic agents

**Figure 5:**
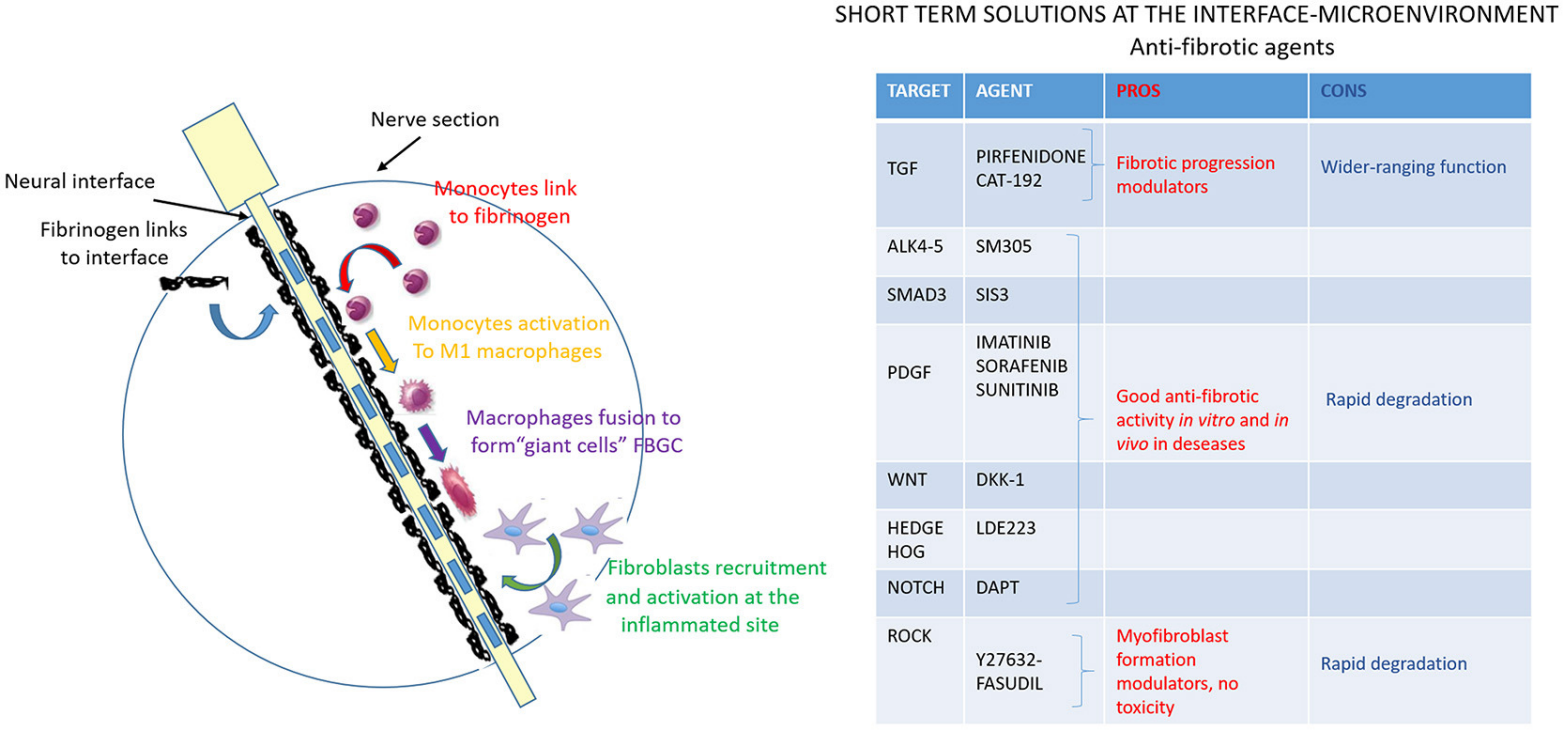
Anti-fibrotic agents to modulate the interface-microenvironment interaction. Schematic image of nerve section implanted with the electrode. The fibrotic process, the last step of FBR, is shown (in green) and represents the fibroblasts recruitment at the implanted inflammation site and their activation to produce extracellular matrix components to form a final dense connective tissue. The table resumes anti-fibrotic agents that can modulate the interface-microenvironment interaction: pros (in red) and cons (in blue) of potential anti-fibrotic drugs targeting mediators of fibrosis are evaluated.

The last step of FBR development is represented by the formation of the fibrotic tissue encapsulating the intraneural interface (Figure 5). Fibrosis is deeply studied (Rosenbloom et al., 2013) due to the wide range of diseases and organ-specific disorders that are characterized by its devastating effects (Schnaper and Kopp, 2003; Bataller and Brenner, 2005; Varga and Abraham, 2007; Cowper, 2008). Although they have a quite different etiology, all the fibrotic processes share common features and mechanisms, leading to the presence of activated fibroblasts or myofibroblasts, expressing the activation marker α-SMA (smooth muscle actin) and producing ECM macromolecules, such as fibrillary type I and type III collagens (Abraham et al., 2007; Krieg et al., 2007). Since the molecular processes involved in fibrosis are very complex and different signaling pathways are activated, a variety of compounds targeting those pathways have been developed as potential anti-fibrotic drugs and they should be considered for their potential effectiveness in FBR context as well.

TGF-β (Transforming Growth Factor-β) signaling is undoubtedly the predominant pathway in fibroblast activation process and its deregulation occurs in essentially all fibrotic reactions. There are two different strategies to act on TGF-β: (i) to directly interfere with its expression and activation, and (ii) to inhibit its receptor and the downstream signaling. Pirfiridone, a TGF-β gene suppressor, has been evaluated in pulmonary (Antoniu, 2006), and in liver fibrosis (Westra et al., 2014), showing its ability to attenuate the fibrotic progression. On the contrary, the clinical trial on CAT-192, the human monoclonal antibody against TGF-β1, has not shown promising results so far. The reason for that can be found in some untoward wide-ranging effects due to the total block of TGF-β (Denton et al., 2007). As for the receptor and pathway inhibitors, there have been several studies on SM305, a specific inhibitor of TGF-β receptors ALK4 and 5, and on SIS3, a specific Smad3 inhibitor (Jinnin et al., 2006). Both of them exert their anti-fibrotic activity *in vitro* and *in vivo*, abrogating TGF-β induced ECM gene expression and fibroblasts transdifferentiation (Ishida et al., 2006).

Imatinib, Sorafenib and Sunitinib are a generation of tyrosine kinase inhibitors that mainly target PDGF (Platelet derived growth factor) pathway, whose inhibition enhances the anti-fibrotic effect of TGF-β blocking drugs. They have been shown to prevent kidney, liver, lung and skin fibrosis formation in several animal models (Daniels et al., 2004; Wang et al., 2005; Yoshiji et al., 2005; Distler et al., 2007; Akhmetshina et al., 2009).

Further interesting kinase in the TGF-β downstream pathway is Rho-associated kinase (ROCK), which plays a role in the actin cytoskeleton reorganization during the activation of fibroblasts into myofibroblasts (Riento and Ridley, 2003; Shimizu et al., 2005). Y27632 (Akhmetshina et al., 2008) and fasudil (Mohri et al., 2003) are two ROCK inhibitors that have been effectively used to block myofibroblasts formation. The absence of cell toxicity makes them good anti-fibrotic candidates.

TGF-β triggers other pro-fibrotic pathways, such as Wnt, Hedgehog and Notch whose inhibitors, respectively, Dkk-1 (Akhmetshina et al., 2012), LDE223, and DAPT are currently under evaluation. Data suggest that Dkk-1, investigated in other diseases, is able to block the pro-fibrotic TGF-β signal; LDE223, evaluated in anti-cancer trial with minimal toxicity, is able to prevent bleomycin-induced dermal fibrosis (Horn et al., 2012) and DAPT is effective on pulmonary and dermal fibrosis in animal model (Kavian et al., 2010; Dees et al., 2011).

It is important to point out that systemic administration of all these anti-fibrotic agents, on one hand, might be not effective due to their rapid degradation and consequent inability to reach a good concentration at the implant site, and, on the other hand, it might raise some safety issues because of their pleiotropic physiological effects. It is very reasonable to think about a topical drug delivery to modulate the local TGF-β pathway activation, since there are already some studies testing this issue on skin fibrosis (Santiago et al., 2005), skin cancer (Mordasky Markell et al., 2010), and corneal fibrosis (Jester et al., 1997). In these studies, the topical use of TGF-β receptor inhibitor or TGF-β blocking antibody showed a remarkable suppression of skin fibrosis via lipogel treatment on shaved skin, a significantly reduction of the fibrotic component of papillomas via injection in the tumor area, and an important modulation of corneal fibrosis via eyewash on the eye. These drugs, even if used in a context different from the intraneural interface field, exert their effects modulating targets of the TGF-β downstream pathway which are common in all types of fibrosis. For the topical administration of TGF-β inhibitors in proximity of implanted electrode, it seems to us very important to evaluate the efficacy of the release perineurally and intraneurally, and to find the best dose to use over time without cytotoxic effects.

## Conclusions and future perspectives

Over the last years, the use of intraneural interfaces has increased in a wide range of applications, such as the control of a new generation of neurally-interfaced prostheses. At present, the success of this technology is limited by an inefficient transduction of the electrical signal, mostly due to the formation of a fibrotic tissue around the implant caused by the FBR. There are many ways by which it is possible to interfere with the FBR, but none of them seems to have solved the issue so far. Starting from recent developments in biomaterials and from novel approaches to inflammatory/fibrotic pathologies, in this review, we have explored and selected the biological solutions that might be adopted in the neural interfaces FBR context.

In particular, we have focused on and provided two main directions to overcome FBR and promote interface stability: acting on the interface properties and acting on the interface-microenvironment interaction. Hitherto, we tried to objectively highlight pros and cons of each approach and, in the following line, we critically state which one, in our opinion, seems the most promising.

Acting on the interface properties, meaning modifying porosity, roughness, stiffness, chemistry with specific surface coatings, is probably the most efficient and feasible strategy, since it modulates the FBR from the initial plasma protein absorption to the interface, the first trigger of the FBR cascade. These coatings are long-term chemically stable, small enough to not alter the surface shape, biocompatible; they can be very easily functionalized and their chemical and electrical properties can be improved. Having the competence and the suitable instrumentation, it is more feasible to modify a material physically and chemically and then directly test it *in vivo*, than having to deal with the choice of a specific target of the complex downstream activated pathway. Indeed, acting on the interface-microenvironment foresees the use of drugs that target the complicated immune system components or the fibroblast cell population. This option, even if doable, requires a more comprehensive evaluation and a more complex set up: i) a good, reproducible *in vitro* model that recapitulates what physiologically happens *in vivo*-so far, there are few 3D *in vitro* model, still not consistent enough, but they might be a starting point to reproduce an immune system cell environment within the nervous system-; ii) the choice of dose and how to specifically deliver any kind of anti-inflammatory/fibrotic substance over the time in order to avoid cytotoxic effects. The choice of a systemic delivery has undesirable long-term and wide-range effects, while topic delivery from specific scaffolds has a limited lifetime. Local release with a tested concentration appears to be a better solution, since it elicits a more specific and less toxic response in the area surrounding the interface. The issue of refilling the scaffold can be overcome by specific technologies for a long-term release, even if it adds more variables to the system. In our opinion, acting on the interface-microenvironment is more difficult to handle and evaluate, is less stable over time, produces more undesirable effects and has less impact on the FBR.

However the possibility of developing a futuristic material to promote electrode bio-integration could open new ways toward the immune response modulation strategy. A promising approach may come from the development of “bio-scaffolds” (Struzyna et al., 2015).

The novelty of this type of scaffolds is the incorporation of living cells (Eberli and Atala, 2006; Korecka et al., 2007), with the main purpose of the creation of a biological interface covering and hiding the electrode, so that the immune system does not recognize it as non-self body (Cullen et al., 2011a,b; Cullen and Smith, 2013) and does not trigger the inflammatory response. The scaffold, placed on an intraneural electrode, can provide a 3D, anisotropic biomaterial structure for different types of cells. A futuristic approach may even see the use of stem cells or autologous nervous cells to avoid host tissue inflammation and over-engineered cells that secrete factors to completely inhibit some FBR driven pathways and promote physiological integration, overcoming the lifetime limitation of soluble factors loaded or coated scaffolds (Eberli and Atala, 2006; Madduri et al., 2010; Rosenstein et al., 2010). Moreover, in case of nerve fiber damage, demyelination and axon retraction, which is a risk that we cannot exclude during the insertion of the intraneural electrode, these scaffolds could secrete trophic factors (Korecka et al., 2007), such as nerve growth factor, glial derived neurotrophic factor, insulin growth factor (Fine et al., 2002; Apel et al., 2010; Madduri et al., 2010; Yan et al., 2012), that mimic the microenvironment, helping cells to restore the damaged nerves (Thompson et al., 2016). Hitherto, in order to create these bio-scaffolds, there are few novel technologies available: (i) 3D printing, that has the advantage of having control over stiffness, pore size and integration of soluble factors and cells more closely resembling the *in vivo* environment (Lee et al., 2014); (ii) “cells electrospinning,” for generation of cell fibers integrated in a scaffold (Arumuganathar and Jayasinghe, 2008); (iii) intraneural electrode with cells electrospinned microchannels coupled with an already existing micro-fluidic technology (Takehara et al., 2014), developed for low invasive biocompatible installation and drug delivery.

The analysis done so far is comprehensive, but the insights we can gather on the successful strategy to overcome intra-peripheral nerve electrode FBR are limited by the following issues: (a) most of the studies on the topic were performed in the CNS; (b) most of the long-term experiments with intraneural electrodes were performed in rats that exhibit different inflammatory response and fibrotic reaction compared to human, also due to the different electrode/nerve size ratio; (c) only one experiment, which lasted <30 days, was done in a large animal (pig); (d) the few implants of intraneural electrodes in human subjects published so far did not established a direct correlation between functional data and FBR. In order to be able to manage intraneural electrodes and deeply understand their real efficacy and clinical applicability, further long-term implants are warranted.

Despite the likely impact of FBR on the long-term implant functionality, it is indeed not possible to estimate the exact incidence or provide statistical data. This is due to the lack of a consistent number of systematical studies involving a coherent group of animals, significant time-points and a reliable general method to measure FBR.

For our scope, how to achieve a reliable measure of FBR is a pertinent topic, but actually, there are no standardized scales; here we summarize the approaches used so far *in vitro* and *in vivo*. *In vitro*, it is possible a direct quantification on fixed tissues from animal model, where macroscopic and microscopic analyses can be made. The general morphological evaluation of the nerve and the capsule surrounding the implant is performed through the histological analysis of the nerve sections, while the grade of inflammatory response is evaluated by immunohistochemistry (IHC) for macrophages and the impact of fibrosis on nerve fiber density by IHC for axons and myelin markers.

Today, only indirect FBR measures have been used *in vivo*, such as the impedance of the electrode contacts which, despite being influenced by multiple factors, correlates with fibrotic tissue development. Indeed, a repeated monitoring of impedance spectrum change can give interesting information on FBR development over time.

Moreover, linked with an impedance increase, FBR alters nerve conduction. Nerve conduction studies provide electrophysiological parameters (amplitude and latency of motor and sensory evoked responses: compound motor action potential-CMAP- and compound nerve action potential-CNAP) that are considered good predictors of an ongoing FBR (Badia et al., 2011; Wurth et al., 2017). In an *in vivo* human application perspective, it would be also very useful to improve some existing non-invasive imaging techniques to gather structural data on FBR, such as echography, with a spatial resolution good enough to discriminate and evaluate the small area around the nerve implant.

A final important consideration is that, compared to other implant devices, neural interfaces have the peculiarity to deliver currents. It is still matter of debate whether this is relevant for FBR development. So far, the only data correlating electrical stimulation with a side effect on surrounding tissues came from brain studies where intracortical stimulating electrodes caused a thicker layer of fibrosis, compared to the non-stimulating ones (Dauth et al., 1977). The size of the lesion, the inflammatory response and the final fibrosis influence local electrical field, charge density and duration of stimulation, due to electrochemical reactions and overheating at the implant site (van Kuyck et al., 2007). However, in a recent publication it has been reported that electrical stimulation does not primarily contribute to enhance the FBR, thus insertion trauma and chemical/mechanical mismatch represent the major players in this process (Wurth et al., 2017). Therefore, even if all the intraneural technology uses a safe range of electrical parameters, thus allowing a prospective clinically-safe stimulation and recording process, it is very important to deeply understand the processes affecting the FBR-stimulation relation. To present knowledge, despite we cannot exclude a peculiar effect of current delivering on post-implantation tissue response, there is not enough scientific evidence to exploit the modulation of the stimulation parameters (waveform and frequency) as an additional strategy to reduce FBR.

## Author contributions

FL designed the paper, analyzed the literature and wrote the paper. FR and GD designed the paper, supervised the writing, wrote and revised the paper. GV, LZ deeply revised the paper. All the authors read and approved the manuscript.

### Conflict of interest statement

The authors declare that the research was conducted in the absence of any commercial or financial relationships that could be construed as a potential conflict of interest.
